# Suprapubic catheterization complicated by an iatrogenic enterocutaneous fistula: a case report

**DOI:** 10.1186/1757-1626-2-9311

**Published:** 2009-12-11

**Authors:** Kushal P Barai, Shahid Islam

**Affiliations:** 1 Department of Urology, Royal Blackburn Hospital, East Lancashire Hospitals NHS Trust, Haslingden Road, Blackburn, BB2 3HH, UK

## Abstract

**Background:**

Suprapubic catheterization is a common procedure employed by urologists to manage long standing voiding dysfunction or neuropathic bladders in patients. Bowel injury is a rare but important complication, in light of the consequences to patient morbidity.

**Case presentation:**

An 81 year old Caucasian female presented with a blocked suprapubic catheter 3 weeks after passage under cystoscopic guidance. The Foley catheter was replaced and attempted aspiration brought up a faeculent fluid. There were no signs to suggest peritonitis. Catheter was kept in situ until computed tomography imaging was obtained; this illustrated the catheter in small bowel with balloon inflated, causing partial small bowel obstruction. Patient underwent exploratory laparotomy with bowel resection, with an uneventful post-operative recovery.

**Conclusion:**

We speculate that the injury occurred at the time of first catheter exchange, with the tip directly piercing the small bowel. To our knowledge this particular mechanism of injury has not previously been reported. This case demonstrates the importance of remaining vigilant to iatrogenic bowel injury after cystostomy, and aids initial management if injury is suspected.

## Background

Suprapubic catheterization is an increasingly common procedure undertaken by Urologists. Indications include recurrent problems passing urethral catheters, urethral trauma, long term incontinence management and suitability for patient [1,2]. The procedure retains a high level of satisfaction among its recipients, but is not without complications [3].

Here we report a rare complication post cystostomy. We believe this case helps to identify those at a particular risk of such an injury, and will serve as an aid to future diagnosis and management of similar episodes. A previously unreported mechanism of injury is also explored.

## Case presentation

An 81 year old Caucasian female, bilateral below knee amputee, presented to the Urology outpatient department with a blocked suprapubic catheter and associated incontinence. She had a longstanding history of urge and stress incontinence.

Suprapubic cystostomy was deemed an appropriate step-up in this patient's voiding dysfunction management. Long term urethral catheterization was proving increasingly difficult and the patient was succumbing to repeated UTIs. The suprapubic catheter was inserted one month prior to current presentation, under direct cystoscopic guidance a 16Ch Foley catheter introduced into the distended bladder, utilising the Lawrence Add-a-Cath approach. (Femcare-Nikomed Ltd, Hampshire, UK.)

No complications were encountered during suprapubic catheter insertion, and the usual precautions were observed [4,5]. Clear yellow urine passed, and continued to pass up until seven days prior to her re-admission on this occasion.

On presentation, other than an uncomfortable fullness sensation there was nil else clinically. There was little by way of resistance when the suprapubic catheter was replaced, which evidently was unsuccessful at re-establishing drainage. Aspiration by syringe was subsequently attempted, and a dark faeculant fluid was aspirated. No further attempt was made to manoeuvre the catheter.

An office ultrasound demonstrated that the newly inserted catheter balloon was inflated, but its location could not be determined. Cystoscopy however ruled out the bladder as the possible site, and interestingly was unable to visualise a scar to suggest recent occupancy of a catheter. The patient had a urethral catheter passed before being admitted, draining a dark-yellow residual distinctly different to the composition of aspirate the suprapubic catheter remained in-situ with the balloon purposefully kept inflated until further investigative imaging could be arranged.

**Figure 1:**
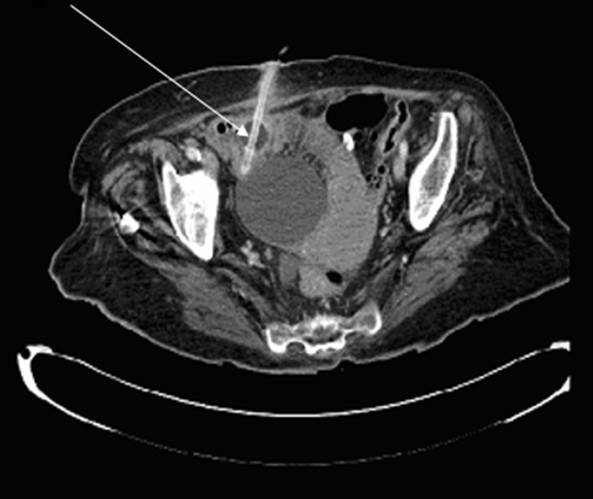
**Axial computed tomography image demonstrating suprapubic catheter in situ of small bowel and balloon inflated causing partial small bowel obstruction (white arrow)**. The bladder appears uninterrupted.

Remarkably the patient remained systemically well and relatively pain free. A mildly distended abdomen apart, there was no overt tenderness or guarding suggestive of peritonitis. Nonetheless a marked rise in blood inflammatory markers, C-reactive protein measuring 53 mg/L and white blood cells 21.8 × 10^9^/L, heightened our concerns. The computed tomography scan confirmed our suspicions of bowel injury the balloon catheter clearly demonstrable in the small bowel, causing partial small bowel obstruction (Figure 1).

Exploratory laparotomy uncovered multiple adhesions attaching the peritoneum of the anterior abdominal wall to bowel viscera. The inflated balloon was palpated within the lumen of the ileum and a fibrosed entry point noted. Oddly no exit point was evident macroscopically, and equally no lesion seen on the bladder. Adhesionectomy, catheter removal, small bowel resection, followed by re-anastomosis concluded surgical intervention. The patient went onto make an uneventful recovery, reverting back to the use of long term urethral catheters.

## Discussion

Frequent postoperative complications of suprapubic catheter insertion include haematuria, urinary tract infections and catheter obstruction [6]. In the case of our patient, a rare presentation of delayed iatrogenic bowel perforation complicated management.

The exact mechanism and order of events which led to this complication is speculative. In the first instance, we can be confident that initial catheter insertion was successful in piercing the bladder, as the procedure was performed under cystoscopic guidance and there was subsequent free drainage of urine. Others have reported piercing of bowel contents en-route to bladder during primary insertion, in effect 'sandwiching' viscera between abdominal wall and bladder, and causing a delayed bowel perforation presentation [7]. Interestingly the authors attribute this occurrence to aberrant anatomy caused by the adhesions. Tilting the patient in a Trendelenburg position utilises gravity to pull bowel contents away from the site of insertion [5]. But this principle cannot hold true if bowel adheres to abdominal wall, as it will remain in the vicinity of catheter passage.

Complete transection is probably far-fetched in our case given only a single defect in bowel was noted, as opposed to an expected entry and exit point.

An alternative theory, possibly the more likely, is that bowel was penetrated directly by the new Foley catheter during catheter exchange in outpatients. The initial catheter may have blocked-up or dislodged by means unknown, rendering it inactive from time of non-drainage. This new tube has then inadvertently pierced the small bowel; adhesions may well have been responsible for positioning abdominal organs along catheter tract course.

Histological analysis of resected bowel segment identified active chronic inflammation along with granulation tissue and fibrosis up to the serosa, consistent with tract formation due to catheter insertion. For our hypothesis to hold, these changes will have taken place in the limited five day timeframe between injury and surgical intervention; quite plausible adjudging by the extensive literature detailing the swiftness of bowel reparation post insult, particularly in small intestine [8]. Besides, the repair process will have employed the inflammatory cascade, and this correlates with the raised inflammation markers after admission.

Of previous reports, there is certainly preponderance to bowel injury at time of initial catheter exchange, usually a month after primary insertion [6]. However, none have so far suggested direct catheter perforation as the mechanism of injury, as appears to be the case here. One might anticipate a threat to organ injury during initial passage, when a manual force is applied to pass the trocar [6]. But it is somewhat surprising the ease with which bowel tissue had been directly penetrated, seemingly by the catheter alone, and without obvious force. This serves a timely reminder that the elderly remain a vulnerable subgroup to such injuries, as tensile strength of visceral tissue declines in age. This group of patients are less likely to demonstrate a virgin abdomen either, and therefore the risk of adhesion associated aberrant anatomy is higher.

This being said, the precise aetiology of bowel perforation is supernumerary to recognising that the event remains a notable risk, and initiating the correct management is imperative to the wellbeing of patients. In our case, choosing not to disturb the catheter after reinsertion appears to have prevented bowel content dissemination into the abdominal cavity. This can very quickly escalate into an overt peritonitic picture entailing significant morbidity and even mortality.

## Conclusion

We present an unusual complication of suprapubic catheter replacement; the formation of an iatrogenic enterocutaneous fistula.

Though a seldom occurrence, it would be prudent of surgeons to remain vigilant of such complications, particular in the aging population. This case highlights that in the event of potential bowel injury, catheter should remain undisturbed until cross sectional imaging has been acquired.

## Abbreviations

UTIs: urinary tract infections.

## Consent

Written informed consent was obtained from the patient for publication of this case report and any accompanying images. A copy of the written consent is available for review by the Editor-in-Chief of this journal.

## Competing interests

The authors declare that they have no competing interests.

## Authors' contributions

KB reviewed the literature, drafted and revised the manuscript. SI was the main surgeon and helped to revise the manuscript. All authors read and approved the manuscript.
